# The Cause and Cure of Pyorrhea Alveolaris by Emetine

**Published:** 1915-01

**Authors:** 


### THE CAUSE AND CURE OF PYORRHEA ALVEOLARIS BY EMETINE.

Last month, on page 1000 of this journal, we printed a portion of an editorial in *The New Orleans Medical and Surgical Journal,* in which reference was made to the work of Bass and Johns of the Tulane College of Medicine, in New Orleans. These investigators have verified the discoveries of Barrett and Smith of Philadelphia relative to the cause of pyorrhea and its cure. The complete article of Bass and Johns, with the discussion, is published in the November number of *The New Orleans Medical and Surgical Journal* (p. 455-456). These gentlemen have identified the organism found in pyorrhea as the *entamœba buccalis.* After a brief introduction, giving the history of this organism and describing the relation of amebæ to other diseases, the authors report the results of their own investigations, as follows:

"We have examined material from the lesions in 87 cases of pyorrhea, and have found amebæ in 85 of them. One of the negative reports was based upon a single slide, prepared at a distance from the laboratory, from the gum of a woman patient who was convalescing from plague. Pus could be squeezed from her gums, and the diagnosis of pyorrhea alveolaris seemed indicated. It may be possible that other preparations from other teeth would have shown amebæ. The other negative case was that of a 13-year-old child with acute gingivitis, involving all the gums, of about eight days' duration. Thorough search failed to show amebæ, and we now believe this probably was not a case of pyorrhea alveolaris.

"In addition to the 86 cases in which the disease was either diagnosed by dentists or was so advanced that no mistake could be made, we have made more than a hundred examinations from apparently normal gums and teeth, either in the mouths of patients who had the disease involving other teeth, or in people who appeared to have normal gum margins. We have not been able to find amebæ in a single such instance. We have, however, found them on several occasions in instances where the gum margin appeared inflamed and diseased, but the best that we could determine the disease did not extend to the alveolar margin. Such cases, perhaps, could not be called pyorrhea alveolaris, but they are probably the beginning of the disease. In several instances we have found amebæ in similarly mildly inflamed and 'easy to bleed' gum margins of one or more teeth in patients who had well-advanced pyorrhea around other teeth. We have found amebæ around teeth in what we have considered to be all the different stages of the disease in the same case, from the slight redness at the gum margin and tendency to bleed easily to the tooth hanging loose in its socket or standing, entirely stripped of gum, on the exposed carious bone.

"The technic of examining for the amebæ is very simple. Remember that they are most numerous in the bottom of the lesion. A little material is removed with a suitable instrument (a toothpick serves the purpose well), diluted on a slide with a little salt solution, saliva (patient's) or water. A cover glass is placed on the diluted material, which should be examined promptly with the high dry lens of the ordinary microscope. By careful search amebæ are found, showing the characteristic ameboid motion. We are not prepared at this time to say whether there is more than one species to be found. The amebæ we have seen vary in size from about that of a leucocyte to about three or four times this size. No contractile vacuole is recognized, but nutritive particles, more refractile and more prominent in appearance, are observed. The ectosarc is quite clear and is well differentiated from the endosarc.

"These amebæ are easily demonstrated in stained specimens. A good method is to make a thin spread of the scrapings and pus from the bottom of the lesion on a slide, allow it to air dry, fix with heat and stain with carbol-fuchsin about one-fourth minute, wash, stain with Loeffler's methylene blue about one-half minute, wash, dry, and examine. The amebæ are well stained by this method, and show their inclusions of tissue or cell remains, indicating pathogenicity. We have been unable to demonstrate that these amebæ take up bacteria, though they sometimes appear to do so.

"Ipecac has been employed with success in the treatment of amebic dysentery for many years, but on account of its nauseating effect and sometimes impossibility for patients to retain it in sufficiently large doses, there has been more or less dissatisfaction in its use. Vedder found that fluid extract of ipecac was destructive to cultural amebæ in solutions of 1 to 200,000. Rogers experimented with the active principle of ipecac, emetine, and found that it would kill entamebæ in stools in solutions of 1 to 100,000, and in 1912 began using it hypodermically in the treatment of amebic dysentery. It is now very generally employed in this manner for this purpose, and with fairly uniform results. The action of emetine in amebic dysentery is very prompt, striking, and specific. Usually the entameba can not be found in the discharges after twenty-four to forty-eight hours of treatment, and the bloody mucous stools give place to normal, formed stools in three or four days. There is considerable tendency to relapse after the treatment has been discontinued for a time, but no doubt a considerable number of 'relapses' are, in fact, reinfections.

"We have not tried the injection of solutions of emetine into the gum and pus pockets, as Barrett and Smith did, because it has not seemed to us reasonably probable that all the diseased tissues could be reached in this way. Whenever a patient has advanced Riggs' disease in one or more teeth, the disease also exists around and between many of the other teeth. The interdental tissue is often soft, spongy, and bleeds readily. Often simply sucking the teeth causes bleeding. Careful examination reveals active motile amebæ present.

"The results of our experiments, so far, are most gratifying. We have had 68 cases under observation and treatment from two days to two weeks. The doses of emetine experimented with have been from one-half to one grain. Only one dose was given in a day. Several cases have been given a dose daily for several days. Others were given one or more doses until the amebæ disappeared, after which an interval was allowed to determine how long it would be before they would return, or what other results could be observed. In several instances no amebæ could be found the next day after the first dose was given. In a few, however, they were found the next day after emetine had been given on two successive days. In no case have we been able to find amebæ the next day after emetine had been given on three successive days.

"As to the duration of the absence of demonstrable amebæ following the three (or less) doses of emetine, our studies have not been conducted long enough to determine. In one instance we found amebæ on the fourth day after the last emetine had been given. In another instance we found them on the sixth day. In several instances none could be found after seven days or longer intermission of treatment. On account of the wide distribution of this ameba in nature and the character of the lesions of the disease, we do not think it very likely that bad cases of pyorrhea alveolaris will be permanently disinfected by a few doses of emetine given during a few days. The chances of reinfection are so great and the damaged gum, alveolar and tooth structure offer such favorable soil, that it must surely be necessary to continue the specific treatment until Nature has had time to fully heal the disease.

"The length of time necessary for this will no doubt depend upon many factors. Healing and repair of diseased bone is always slow. Whenever the disease involves only the gum, and has not reached the bone (alveolar structure), it is our impression from observances so far made, that probably the length of time necessary for the gum to heal will not exceed a week. We have observed great change in forty-eight hours, and gums that bleed easily often become perfectly normal in this regard in from twenty-four to seventy-two hours. The results are so striking that there is no doubt in the mind of the doctor or the patient.

"Our experiments have not advanced sufficiently to enable us to lay down dogmatic rules as to the treatment, but we are certain that rapid and favorable results may be expected to follow the administration of 1-2 grain of emetine hydrochloride hypodermatically (in any part of the body) daily for three or four days. In all except the early, mild cases it may be necessary to repeat the treatment, during one or more days, after an interval of three to ten days. In the worst cases no doubt it will be found necessary to repeat the treatment several times before the disease is entirely well. No doubt removal of tartar, scales and other local dental treatment should also be done at the same time.

"It is quite likely that the injection of a weak solution of emetine, one-half per cent., as used by Barrett, into such lesions as can be reached by it, will be found to favor success from the hypodermic treatment with emetine."

—*Clinical Medicine.*

